# Identification of the High-Affinity Potassium Transporter Gene Family (HKT) in *Brassica* U-Triangle Species and Its Potential Roles in Abiotic Stress in *Brassica napus* L.

**DOI:** 10.3390/plants12213768

**Published:** 2023-11-04

**Authors:** Xiaoran Yang, Ran Hu, Fujun Sun, Shulin Shen, Mengzhen Zhang, Yiwei Liu, Yi Zhang, Hai Du, Kun Lu, Cunmin Qu, Nengwen Yin

**Affiliations:** 1 Integrative Science Center of Germplasm Creation in Western China (CHONGQING) Science City, College of Agronomy and Biotechnology, Southwest University, Chongqing 400715, China; yangxiaoran2003@163.com (X.Y.); hr1996@email.swu.edu.cn (R.H.); drsunfujun@email.swu.edu.cn (F.S.); ssl7159@email.swu.edu.cn (S.S.); zmz0110@email.swu.edu.cn (M.Z.); liuyiwei1942@outlook.com (Y.L.); 15723322247@163.com (Y.Z.); haidu81@126.com (H.D.); drlukun@swu.edu.cn (K.L.); 2 Academy of Agricultural Sciences, Southwest University, Chongqing 400715, China; 3 Affiliation Engineering Research Center of South Upland Agriculture, Ministry of Education, Chongqing 400715, China

**Keywords:** high-affinity K^+^ transporter (HKT), triangle of U, *Brassica napus* L., abiotic stress, heavy-metal tolerance

## Abstract

Members of the high-affinity potassium transporter (HKT) protein family regulate the uptake and homeostasis of sodium and potassium ions, but little research describes their roles in response to abiotic stresses in rapeseed (*Brassica napus* L.). In this study, we identified and characterized a total of 36 *HKT* genes from the species comprising the triangle of U model (U-triangle species): *B. rapa*, *B. nigra*, *B. oleracea*, *B. juncea*, *B. napus*, and *B. carinata*. We analyzed the phylogenetic relationships, gene structures, motif compositions, and chromosomal distributions of the HKT family members of rapeseed. Based on their phylogenetic relationships and assemblage of functional domains, we classified the HKT members into four subgroups, HKT1;1 to HKT1;4. Analysis of the nonsynonymous substitutions (Ka), synonymous substitutions (Ks), and the Ka/Ks ratios of *HKT* gene pairs suggested that these genes have experienced strong purifying selective pressure after duplication, with their evolutionary relationships supporting the U-triangle theory. Furthermore, the expression profiles of *BnaHKT* genes varies among potassium, phytohormone and heavy-metal treatment. Their repression provides resistance to heavy-metal stress, possibly by limiting uptake. Our results systematically reveal the characteristics of HKT family proteins and their encoding genes in six *Brassica* species and lay a foundation for further exploration of the role of *HKT* family genes in heavy-metal tolerance.

## 1. Introduction

Plant growth, development, and productivity are negatively affected by multiple abiotic stresses, including heavy metals and salinity, due to the unfavorable conditions they impose. In response, plants have developed compensatory systems allowing them to adjust to abiotic stress conditions through a complex cascade of specific physiological, molecular, and cellular adaptations [1]. The potassium cation (K^+^) is an essential plant nutrient and plays crucial roles in response to a variety of abiotic stresses such as salt stress, drought stress, oxidative stress, and so on [2,3]. Potassium transporters form several protein families: the KT (K^+^ transporter)/HAK (high-affinity K^+^)/KUP (K^+^ uptake) family, the Trk (K^+^ transporter)/HKT family, the KEA (K^+^ efflux antiporter) family, and the CHX (cation/hydrogen exchanger) family [4,5]. Of these, the Trk/HKT family occurs only in plants [6], and mainly regulates the uptake and homeostasis of sodium and potassium ions [7].

To date, numerous studies have identified *HKT* genes in many monocots and dicots, including wheat (*Triticum aestivum* L.) [8,9,10,11], Arabidopsis (*Arabidopsis thaliana*) [9,11,12,13], rice (*Oryza sativa* L.) [9,14,15], barley (*Hordeum vulgare* L.) [9,11,16], maize (*Zea mays* L.) [9,15], tomato (*Solanum lycopersicum* L.) [9,17], sorghum (*Sorghum bicolor*) [9,18], soybean (*Glycine max*) [9,19], foxtail millet (*Setaria italica*) [20], and some *Rosaceae* species [9]. HKTs share a characteristic structure made up of four transmembrane domain-pore domain-transmembrane domain units (MPM1-MPM4) [7]. Each unit consists of one pore-like region (P) and two transmembrane regions (M) [21].These *HKT* genes are categorized into two different subfamilies (class I and class II) based on the structure of the first pore loop of the protein they encode, which determines their transport selectivity [22]. Generally, class I HKTs (with the sequence S-G-G-G in the first pore loop) function as sodium uniporters, whereas class II HKTs (with G-G-G-G in the first pore loop) are Na^+^/K^+^ symporters [6,7]. For example, studies in Arabidopsis have shown that certain *HKT* gene family members can remove excessive Na^+^ from the xylem under salt stress, protect plants against damage to photosynthetic cells, and play an important role in Na^+^ excretion and K^+^ homeostasis in leaves [11,13,23].

*Brassica* species include numerous vegetable and oil crops of global importance, of which six economically important species are known as U-triangle species: *B. rapa*, *B. nigra*, *B. oleracea*, *B. juncea*, *B. napus*, and *B. carinata* [24,25]. The three ancestral diploid species are *B. rapa*, *B. nigra* and *B. oleracea* (*B. rapa*, the AA subgenome donor; *B. nigra*, the BB subgenome donor; *B. oleracea*, the CC subgenome donor) and three derived allotetraploid species are *B. juncea*, *B. napus*, and *B. carinata* (*B. juncea*, harboring AABB; *B. napus*, carrying AACC; and *B. carinata*, with BBCC). Importantly, the genetic relationships of U-triangle species have been clarified based on a comprehensive genomics approach, providing important genomic resources for correlative and evolutionary studies [24]. Moreover, many gene families have been identified and characterized in these *Brassica* U-triangle species, including genes encoding cytokinin response factors (CRFs) [26], auxin response factors (ARFs) [27,28], and mitogen-activated protein kinases (MAPKs) [29]. However, the *HKT* gene family in *Brassica* species has not been well characterized, and there has been a lack of systematic study until now.

Rapeseed (*Brassica napus* L.) is one of the most important oilseed crops worldwide, with many agronomic advantages, such as rapid growth, high biomass productivity, and strong adaptability to diverse environmental conditions. Recent studies show that many protein families in rapeseed have potential functions to respond to salt and heavy-metal stresses, including cysteine-rich polycomb-like proteins (CPP) [30], homeodomain-leucine zipper proteins (HD-Zip) [31], heavy-metal ATPases (HMA) [32], and phosphorus transporters (PHT) [33]. *B. napus* is a moderately salt-tolerant species [34], and is a known heavy-metal accumulator [35]. Moreover, the germplasm resources of *B. napus* are rich in genetic diversity and contain a variety of excellent genes that can be used for the remediation of soil contaminated with salts and heavy metals. *HKT* genes encode important ion transporters in plants, which can improve stress tolerance by regulating sodium and potassium transportation and concentration in plants [7,13,36]. Therefore, characterization of the *HKT* genes in *B. napus* (*BnaHKTs*) and analysis of adaptive mechanisms in stress-tolerant varieties has far-reaching significance for the valorization and remediation of contaminated soil. In the current study, we identified 36 *HKT* genes in the genomes of U-triangle species and comprehensively analyzed features of their sequences, their chromosomal locations, and the phylogenetic relationships of their encoded proteins. We also investigated the expression patterns of *BnaHKT* genes in response to abiotic stress to identify their functions. Our results lay an important foundation for further understanding the regulation of *HKT* expression in *B. napus* under abiotic stress and provide avenues for the improvement of this globally important crop.

## 2. Results

### 2.1. Identification of HKT Family Genes in the Brassica U-Triangle Species

We identified a total of 38 putative HKT proteins in the six *Brassica* U-triangle species: *B. rapa* (Bra), *B. nigra* (Bni), *B. oleracea* (Bol), *B. juncea* (Bju), *B. napus* (Bna) and *B. carinata* (Bca). However, we eliminated BniB08g052450.2N and Bol007717 due to their incomplete structures. We thus retained 36 putative HKT proteins, consisting of five BraHKTs, four BniHKTs, three BolHKTs, eight BjuHKTs, eight BnaHKTs and eight BcaHKTs, for analysis (Table 1). We renamed these HKT members as HKT1;1 to HKT1;4: the first number behind HKT represented class I or II, and the second number behind HKT corresponds to the classified subfamilies in this study, with the two numbers separated by a semicolon (;). The characteristics of the encoded HKT proteins are listed in Table 1 (gene ID, protein size, molecular weight (MW), isoelectric point (pI), and predicted subcellular location). The lengths of the proteins ranged from 350 to 585 amino acid residues, and their MWs varied from 39.60 to 65.84 kDa. The pIs of all of the HKTs were greater than 7, ranging from 8.84 to 9.76. Subcellular localization predictions revealed that nearly all of the family members may localize to cell membranes, with BcaHKT1;2a being the only one predicted to localize to chloroplasts.

### 2.2. Phylogenetic Analysis of HKT Proteins in Arabidopsis and the Brassica U-Triangle Species

**Figure 1:**
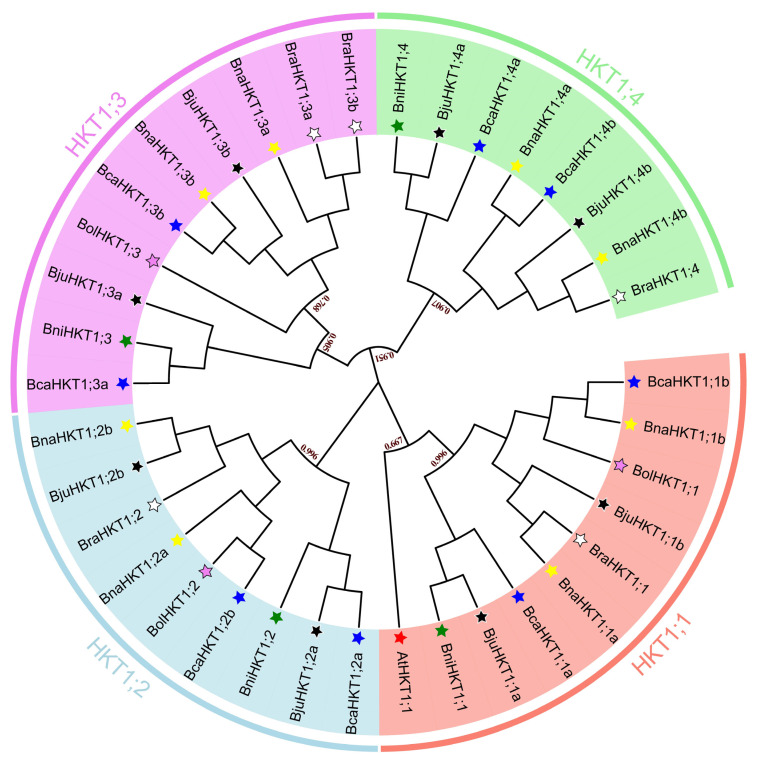
Phylogenetic tree of the HKT protein family from Arabidopsis and the *Brassica* U-triangle species. The HKT family was divided into four subgroups (HKT1;1–4), which are shown in red, blue, purple and green for HKT1;1–4, respectively. Arabidopsis, red star; *B. napus*, yellow star; *B. rapa*, white star; *B. juncea*, black star; *B. oleracea*, purple star; *B. carinata*, blue star; *B. nigra*, green star. For renaming, a species-specific prefix is included, and a lowercase letter suffix is used to represent the gene number within each clade. The dark red numbers beside the main nodes are bootstrap scores.

The HKT family members of the U-triangle species all belong to subfamily I (with the S-G-G-G sequence) [7]. To reveal the evolutionary relationships among the HKT family members, we reconstructed a phylogenetic tree using protein sequences from Arabidopsis AtHKT1;1 and the 36 proteins from the *Brassica* U-triangle species. Based on their relative positions in the tree and their functional domains, we classified all proteins into one of four subgroups: HKT1;1 to HKT1;4 (Figure 1). HKT1;3 and HKT1;4 members were located in the same larger phylogenetic branch. The HKT1;1 subgroup contained sequences from all seven species, while the HKT1;2 and HKT1;3 subgroup included members of all six U-triangle species, but not Arabidopsis. HKT1;4 members were present in *B. nigra*, *B. juncea*, *B. carinata*, *B. napus* and *B. rapa*. The number of members in the four subgroups is approximately equal, with 10 in HKT1;1 (including AtHKT1;1), nine in HKT1;2, 10 in HKT1;3 and eight in HKT1;4. The phylogenetic tree had clear and reliable clusters, and adjacent sequences within the same clade had close evolutionary relationships, consistent with the U-triangle pattern between the allotetraploids (*B. napus*, *B. juncea*, and *B. carinata*) and their diploid progenitors (*B. rapa*, *B. oleracea*, and *B. nigra*). 

### 2.3. Multiple Sequence Alignment of HKT Proteins in the U-Triangle Species

**Figure 2:**
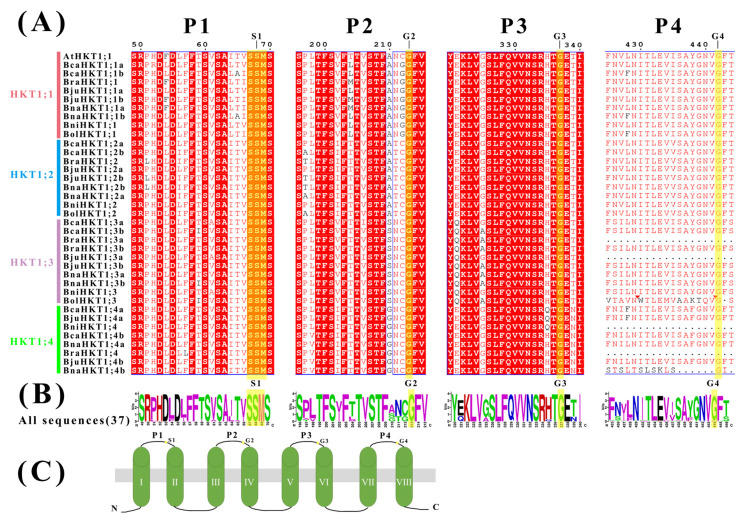
Sequence analysis of HKT family members in Arabidopsis and the *Brassica* U-triangle species. (**A**) Sequence alignment of the conserved pore domain is shown in the figure, and P1–P4 indicate the four pore domains. The red triangles indicate the locations of sequences that are less conserved. The yellow shading represents the crucial residues that were used for classification. The subgroups are labeled in the same colors as in Figure 1. (**B**) WebLogos analysis of the conservation at the S1 and G2 amino acid residue pore-forming regions. (**C**) The transmembrane helices are labeled I to VIII with the conserved residues (S-G-G-G).

To visualize the conserved domains in the HKT protein sequences, we performed a multiple sequence alignment using AtHKT1;1 from Arabidopsis, and 36 HKT protein sequences from the six *Brassica* U-triangle species. Using NCBI and Interpro conserved domain analysis, we determined that all identified HKT family members contain TrkH [37] and 2a38euk (https://www.ncbi.nlm.nih.gov/Structure/cdd/TIGR00934 (accessed on 26 November 2022)) [38] conserved domains, although the number and position of the domains varied. DeepTMHMM detected eight distinct transmembrane helices in all HKT proteins, except for BcaHKT1;2a, BraHKT1;3a, BraHKT1;4, BjuHKT1;3a, BnaHKT1;4b, BniHKT1;4, BolHKT1;3, which have seven, six, six, six, seven, six, and seven helices, respectively (Figure 2C, I–VIII). 

Proteins in the HKT family have a characteristic structure containing four loops forming the pore [7,11]. The members of the HKT family can be classified into subfamily I and subfamily II based on the residues in the first pore loop that form the selectivity filter—a Ser residue (S-G-G-G) for subfamily I and a Gly residue (G-G-G-G) for subfamily II [7]. As dicotyledons, U-triangle species only have subfamily I HKTs [7,11]. The presence of a Ser residue supported this idea (Figure 2A). There are four conserved selectivity filter–pore regions (P-loops: P1 to P4, Figure 2). Among the four pore domains, P4 was less conserved than the other three. BraHKT1;3a, BjuHKT1;3a, BniHKT1;4 and BraHKT1;4 lacked P4, while BnaHKT1;4b and BolHKT1;3 lacked some fragments in P4 but still contained the Gly residue (Figure 2). These characteristic sequences may have been lost during evolution. Even so, the pore regions for the *Brassica* species are highly conserved, confirming that the species share high similarity.

### 2.4. Conserved Protein Motifs of HKT and Variations in Gene Structure

**Figure 3:**
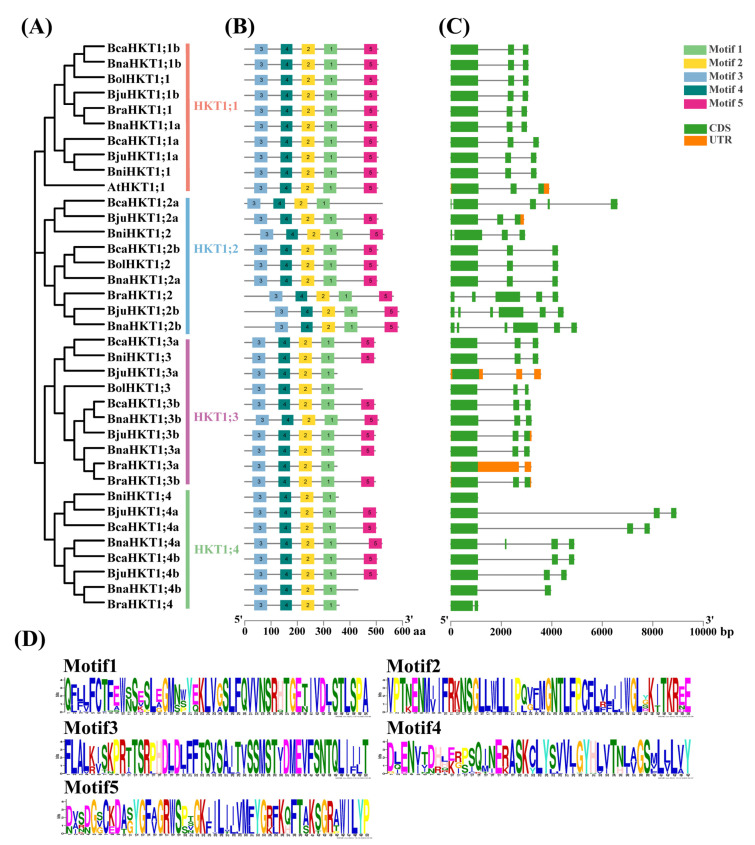
Phylogenetic tree, motif distributions, and gene structure analysis of HKTs between Arabidopsis and the U-triangle species. The conserved motif map and gene structure map are shown alongside the phylogenetic tree. (**A**) Phylogenetic tree of the HKT protein family from Arabidopsis and the *Brassica* U-triangle species (same with Figure 1). (**B**) Conserved motifs of the HKT proteins. Five motifs were identified using the MEME program, and are indicated as differently colored boxes. The black lines indicate relative protein lengths. (**C**) Gene structure of *HKT* family genes. Coding sequences (CDS), green boxes; introns, gray lines; untranslated regions (UTRs), orange boxes. (**D**) The sequences of the 5 Motifs for Figure 3B. Each subgroup on the left side is labeled in the same color as in Figure 1.

To further investigate relationships among the HKT family members in each subgroup, we identified their conserved motifs and corresponding gene structures (Figure 3). We detected five conserved motifs among the 37 HKT proteins between Arabidopsis and the U-triangle species. All HKT members contained motifs 1 to 4. The first conserved residue (Ser) was in Motif 3, and the second conserved residue (Gly) in Motif 2. The third conserved residue (Gly) was in Motif 1, and the fourth conserved residue (Gly) was between Motif 1 and Motif 5 (Figure 3).

The exon and intron structure of *HKT* genes was more complex. *BjuHKT1;2b* and *BnaHKT1;2b* contained the most exons (six), while *BraHKT1;3a*, *BjuHKT1;3a* and *BniHKT1;4* had the fewest exons (one). The number of exons varied among members of the HKT family with diversity gene structures (Figure 3). However, the numbers of exons and exon–intron patterns were similar within each HKT family subgroup, supporting the notion that our classification method was reliable.

### 2.5. Chromosomal Distribution of HKT Genes in the U-Triangle Species

**Figure 4:**
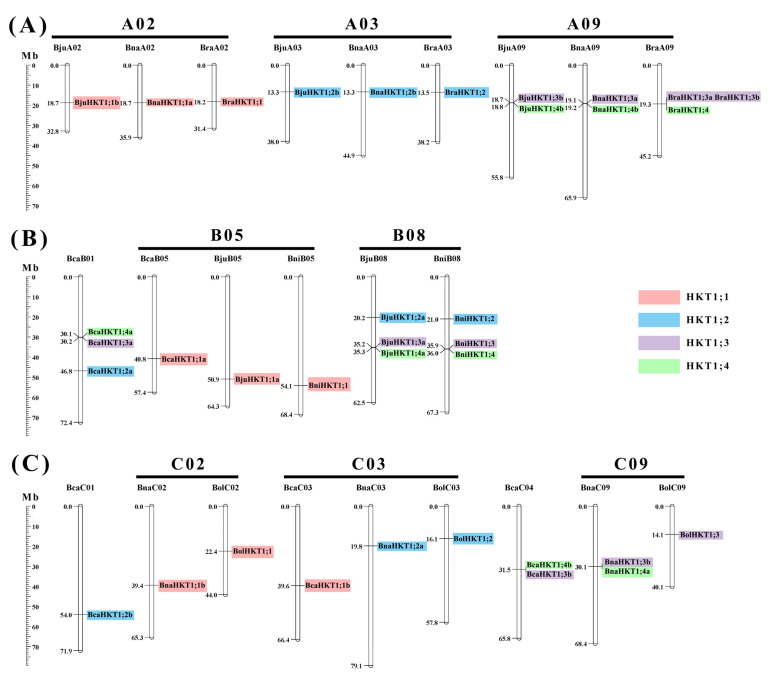
Chromosome localization of *HKT* genes across U-triangle species. (**A**) *HKT* genes located on the A subgenome in *B. juncea*, *B. napus*, and *B. rapa*. (**B**) *HKT* genes located on the B subgenome in *B. juncea*, *B. carinata*, and *B. nigra.* (**C**) *HKT* genes located on the C subgenome in *B. napus*, *B. carinata*, and *B. oleracea*. The distributions of the *HKT* genes are in accordance with the evolutionary relationships in U-triangle species. The labels above the corresponding chromosomes indicate the name of the source organisms and their subgenomes. The scales to the left indicate the sizes of the various *Brassica* chromosomes in Mb. Genes in the same subgroup are shaded using the same colors as in Figure 1.

In total, the 36 *HKT* genes mapped onto 24 chromosomes of U-triangle species, with chromosomes in the A (13 genes), B (12 genes), and C (11 genes) subgenomes, which are unevenly distributed (Figure 4). Moreover, *HKT1;3* and *HKT1;4* are closely associated; some even belong to the tandem duplication event (Figure 4). We also found that *HKTs* in the same subgenome were generally located in parallel physical positions, suggesting that extensive collinearity existed among them in U-triangle species. In addition, some *HKT* family genes were just detected in *B. carinata*, suggesting that duplicated genes have been lost during evolution.

### 2.6. Collinearity Analysis of HKT Genes in U-Triangle Species

**Figure 5:**
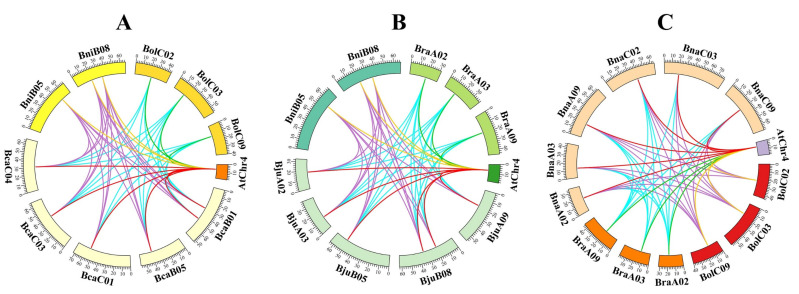
Collinearity analysis of *HKT* family genes between Arabidopsis and U-triangle species. (**A**) Collinearity analysis of *HKT* family genes among Arabidopsis, *B. nigra*, *B. oleracea*, and *B. carinata*. (**B**) Collinearity analysis of *HKT* family genes among Arabidopsis, *B. nigra*, *B. rapa*, and *B. juncea*. (**C**) Collinearity analysis of *HKT* family genes among Arabidopsis, *B. oleracea*, *B. rapa*, and *B. napus*. AtChr4 is the only chromosome with one *HKT* gene in Arabidopsis. The chromosomes are shown in different colors from the U-triangle species. The different colored lines represent the syntenic regions. The scales represent the length of the chromosomes.

Arabidopsis, the allotetraploids (*B. napus*, *B. juncea*, and *B. carinata*) and their diploid progenitors (*B. rapa*, *B. oleracea*, and *B. nigra*) were divided into three groups according to the subgenome relationships, respectively, from which we identified orthologous gene pairs (Figure 5). Group A comprised Arabidopsis and *B. nigra* (three pairs of orthologous genes), Arabidopsis and *B. carinata* (six pairs), Arabidopsis and *B. oleracea* (three pairs), *B. nigra* and *B. carinata* (18 pairs), and *B. oleracea* and *B. carinata* (16 pairs). Group B contained Arabidopsis and *B. nigra* (three pairs of orthologous genes), Arabidopsis and *B. juncea* (six pairs), Arabidopsis and *B. rapa* (three pairs), *B. nigra* and *B. juncea* (16 pairs), and *B. rapa* and *B. juncea* (18 pairs). Group C consisted of Arabidopsis and *B. oleracea* (three pairs of orthologous genes), Arabidopsis and *B. napus* (six pairs), Arabidopsis and *B. rapa* (three pairs), *B. oleracea* and *B. napus* (16 pairs), and *B. rapa* and *B. napus* (17 pairs). Most *HKT* family genes in three allotetraploid species showed the higher collinearity with Arabidopsis and their diploid progenitors, except for nine *HKT* family genes, including *BcaHKT1;3a*, *BcaHKT1;3b*, *BniHKT1;4*, *BraHKT1;4*, *BraHKT1;3a*, *BjuHKT1;4b*, *BjuHKT1;4a*, *BnaHKT1;4a*, and *BnaHKT1;4b*. 

We calculated the numbers of nonsynonymous substitutions (Ka), synonymous substitutions (Ks), and the Ka/Ks ratios for the *HKT* gene pairs to reveal the evolutionary constraints acting on the *HKT* gene pairs. Among the 563 gene pairs compared, only *BcaHKT1;1b* and *BolHKT1;1*, *BnaHKT1;1b* and *BolHKT1;1* had Ka/Ks ratios > 2, while the Ka/Ks ratios of other gene pairs were all below 1, with most of the values ranging between 0.1 and 0.3. This observation suggested that the *HKT* family genes in the seven species have experienced strong purifying selective pressure following duplication. 

### 2.7. Expression Profiles of HKT Family Genes in B. napus under Potassium and Phytohormone Treatment

**Figure 6:**
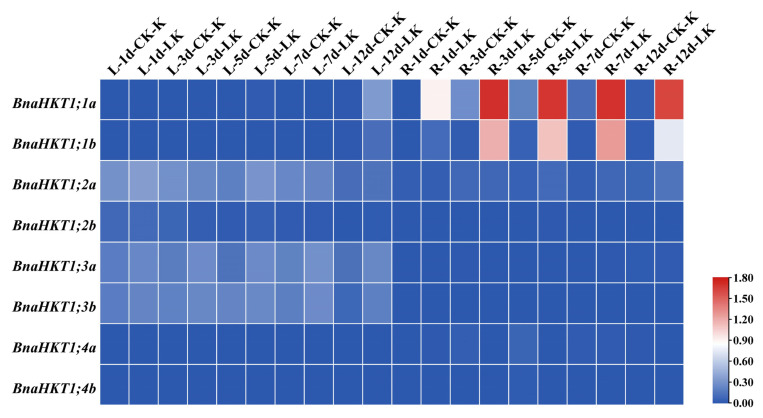
Expression profiles of *BnaHKTs* under potassium treatment. The expression profiles of each *BnaHKT* were calculated as Log_10_ (FPKM value + 1). L, leaves; R, roots; d: days; LK, low-potassium treatment; CK (control) indicates normal nutrient solution with no potassium deficiency. FPKM, fragments per kilobase of exon model per million.

Based on the published RNA-Seq data (http://www.bnagadb.cn/), we examined the expression of the *HKT* genes in rapeseed was affected by abiotic stresses, including the different potassium concentrations and different phytohormones (IAA, indole-3-acetic acid or heteroauxin; ACC, 1-aminocyclopropanecarboxylic acid; ABA, abscisic acid; GA3, gibberellin; 6-BA, *N*-6-benzyladenine), respectively. Overall, *BnaHKT1;1a* and *BnaHKT1;1b* were most significantly induced upon low potassium conditions in roots (Figure 6). *BnaHKT1;2a*, *BnaHKT1;3a* and *BnaHKT1;3b* displayed the highest expression level in leaves among both control (CK) groups and low potassium (LK)-treated groups, and *BnaHKT1;3a* and *BnaHKT1;3b* were significantly upregulated when subjected to a low potassium concentration. The *HKT* genes were upregulated, presumably to counteract potassium deficiency by raising the uptake of this element, suggesting that the different *BnaHKTs* have distinct roles in different tissues.

**Figure 7:**
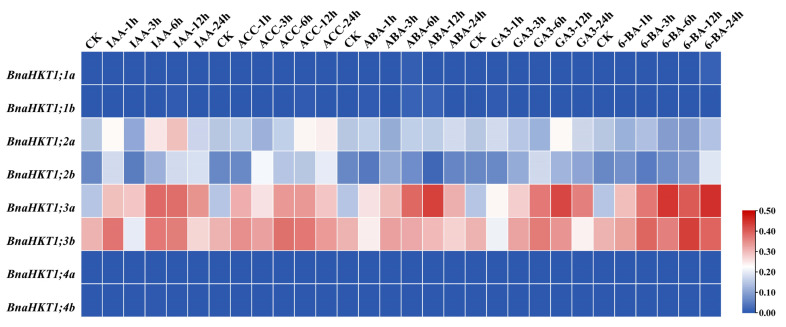
Expression profiles of *BnaHKTs* under phytohormone treatments. The expression profiles of the *BnaHKTs* were calculated as Log_10_ (FPKM value + 1). IAA, indole-3-acetic acid or heteroauxin; ACC, 1-aminocyclopropanecarboxylic acid; ABA, abscisic acid; GA3, gibberellins; 6-BA, 6-benzyladenine. CK (control) indicates normal nutrient solution with no phytohormone treatments. Labels 1 h, 3 h, 6 h, 12 h, and 24 h represent the hours after treatment.

To well understand the phytohormone-induced expression profiles of the *BnaHKTs*, we investigated their expression levels in seeding roots of ZS11 under different phytohormone treatments. Notably, *BnaHKT1;2b*, *BnaHKT1;2a*, *BnaHKT1;3a* and *BnaHKT1;3b* were sensitive to phytohormones, and showed the similar trend (Figure 7). Generally, for IAA and ACC treatment, we observed a significant downregulation between 1 and 3 h and an upregulation between 3 and 12 h, and another downregulation during 12–24 h (Figure 7). However, expression under ABA, GA3 and 6-BA treatments followed a unimodal pattern, with a decrease in expression after reaching a peak during 3–24 h. Based on the variation of transcript levels, we suggest that *BnaHKT1;3a* and *BnaHKT1;3b* should play crucial roles in response to phytohormones, but the members of *BnaHKT1;1a*, *BnaHKT1;1b*, *BnaHKT1;4a* and *BnaHKT1;4b* showed less sensitivity to phytohormones treatments. In conclusion, *BnaHKTs* had a variety of responses to treatments with several phytohormones. 

### 2.8. Expression Patterns of HKT Genes in Rapeseed under Heavy-Metal Stress

**Figure 8:**
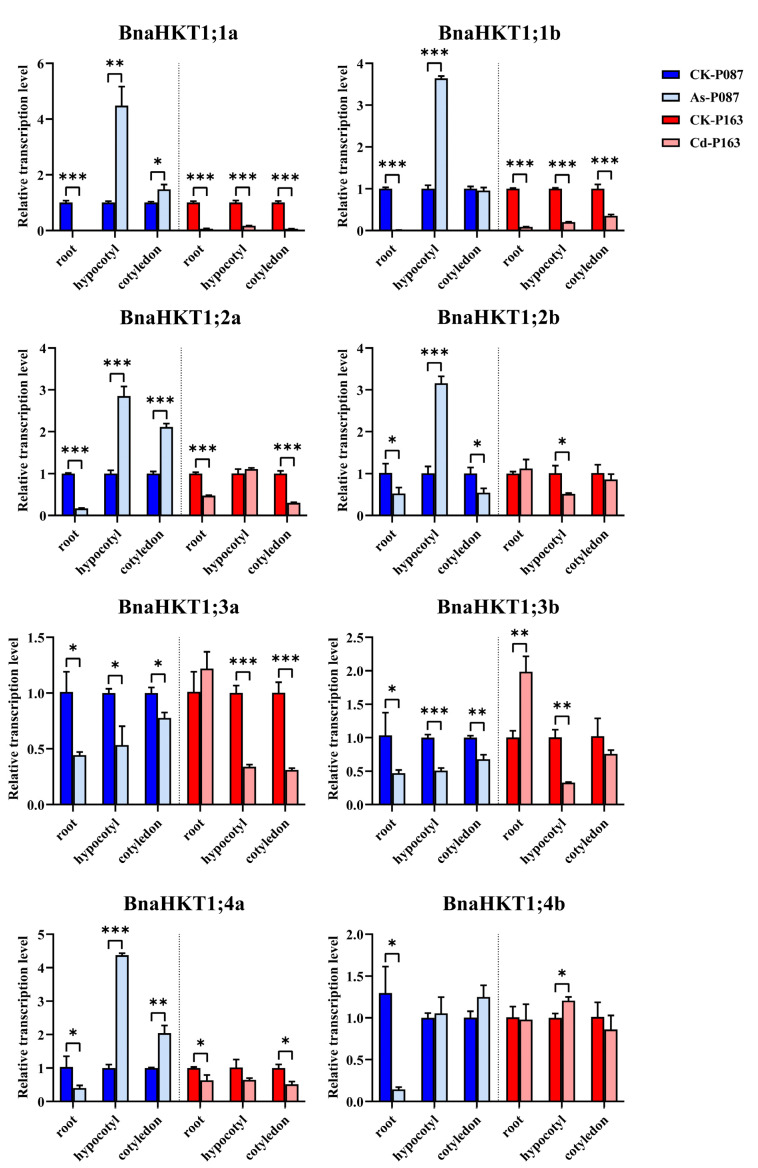
Expression patterns of *BnaHKTs* under As^3+^ or Cd^2+^ treatment. Data obtained by RT-qPCR were normalized to the expression level of *BnaActin7*, and error bars represent the standard deviation (SD) of three biological replicates Statistically significant differences were analyzed by Student *t*-test. * *p* < 0.05; ** *p* < 0.01; *** *p* < 0.001. P087 and P163 are the two accessions used for heavy-metal treatment.

To reveal the possible functions of *BnaHKT* genes in response to heavy-metal stress in *B. napus* seedling, we detected the expression profiles of eight *BnaHKTs* under heavy-metal stress by RT-qPCR analysis in roots, hypocotyls, and cotyledons, respectively. As shown in Figure 8, these members showed the variation expression patterns. Under As^3+^ treatment, all *BnaHKTs* were repressed in roots and *BnaHKT1;3a*, *BnaHKT1;3b* and *BnaHKT1;4b* were inhibited in different tissues, while *BnaHKT1;1a*, *BnaHKT1;1b*, *BnaHKT1;2a*, *BnaHKT1;2b* and *BnaHKT1;4a* showed the highest expression levels in hypocotyls (Figure 8). Under Cd^2+^ treatment, all *BnaHKTs* were obviously downregulated, including *BnaHKT1;1a*, and *BnaHKT1;1b* in roots, hypocotyls, and cotyledons; *BnaHKT1;2a* and *BnaHKT1;4a* in roots and cotyledons. Our findings indicate that these *BnaHKTs* showed the different expression levels in response to heavy-metal stress, suggesting that they may have potential functions in protection from heavy-metal stress.

### 2.9. Cis-Element Analysis of BnaHKT Promoters

**Figure 9:**
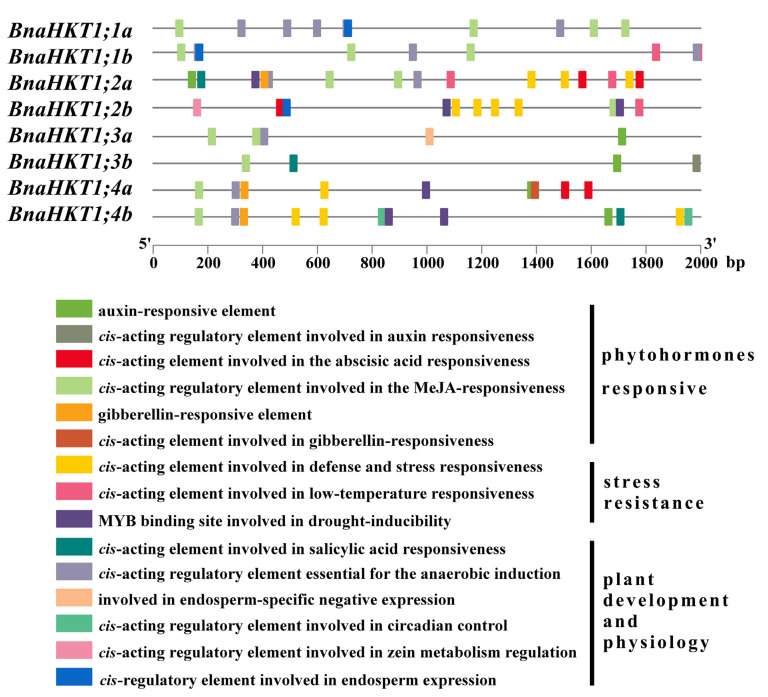
Predicted *cis*-elements in *BnaHKT* promoters. The analysis was based on the 2000-bp regions 5′ to *BnaHKT* transcription start sites. The *cis*-acting elements identified are marked with differently colored boxes, and the functional categories are noted at the right.

We carried out *cis*-element analyses in the 2000 bp regions upstream of the *BnaHKT* gene transcription start sites, and identified several fundamental *cis*-acting elements related to hormone, plant development and abiotic responses. In this study, each *BnaHKT* promoter contained the light-responsive elements (LRE) [39] and *cis*-acting regulatory element involved in the MeJA (methyl jasmonate response element) responsiveness [40]. Moreover, in *BnaHKT* promoters, our predictions revealed six types of phytohormone-responsive *cis*-elements (including ABA, auxin and GA-responsive elements), three types of stress-resistance *cis*-elements (defense, drought-inducibility, and low-temperature responsiveness) and six types of *cis*-elements involved in plant growth and development (Figure 9). These data indicate that *BnaHKT* genes play important roles in stress resistance, plant biology and hormone signaling pathways.

## 3. Discussion

The HKT family is a plant-specific protein family that is believed to control a variety of developmental and stress responses in plants [6,7]. Previous studies showed that the *HKT* family genes regulate K^+^ concentration and homeostasis in plants [7,36]. To date, the HKT family has been identified in different species [8,9,10,11,12,14,15,16,17,18,19,20]. In this study, we identified 36 HKT family members in the six *Brassica* U-triangle species. Although divergences were found among the number of *HKT* genes in *Brassica* species, the number of *HKT* genes in *B. juncea* (AABB, 8), *B. napus* (AACC, 8), and *B. carinata* (BBCC, 8) was roughly equal to the sum of those in their corresponding diploid ancestors *B. rapa* (AA, 5) plus *B. nigra* (BB, 4), *B. rapa* (AA, 5) plus *B. oleracea* (CC, 3), and *B. nigra* (BB, 4) plus *B. oleracea* (CC, 3), respectively. In addition, based on our analysis of Ka/Ks values, most of the *HKT* genes experienced strong purifying selective pressure during evolution, and they retained a high degree of collinearity along their respective chromosomes after diverting from the same ancestor.

Previous studies have shown that HKT family proteins can be categorized as class I and class II according to the presence of different functional residues [7]. Moreover, HKT members in dicots contain only subgroup I (S-G-G-G) [7]. In agreement, all *HKT* genes in the six *Brassica* species were all assigned to subgroup I (S-G-G-G) (Figure 2.). In this study, we also categorized the HKT family members into four subgroups (HKT1;1 to HKT1;4) based on their evolutionary relationships and reconstructed a phylogenetic tree. Located in the same larger phylogenetic branch, members in HKT1;3 and HKT1;4 are more closely related to each other than to members of other subgroups. The HKT family members showed a higher similarity in motif pattern, gene structure, parallel physical positions within the same subgroups, and variation between the different branches and members. In addition, we detected Motifs 1, 2, 3, and 4 in all HKT family members, suggesting that these four motifs represent the functional domains that play ion transport roles in the HKT family, a result consistent with previous studies [41]. By contrast, Motif 5 was lacking in some of the family members but might still retained some properties as HKT proteins. These results suggest that, in each subgroup, *HKT* family genes might have experienced the same evolutionary pressures and have similar functions. 

In modern agricultural practices, abiotic stresses are a major constraint to crop production [42]. HKT family members have many functions, including Na^+^ and K^+^ uptake and Na^+^-K^+^ homeostasis [7,13]. Our results confirmed that the expression of *HKT* genes was induced under low K^+^ concentration, corroborating the basic function of this family under high-salinity conditions. In this study, *BnaHKT1;1a* and *BnaHKT1;1b* were induced by low-K^+^ (Figure 6), suggesting that they may play positive roles in regulating potassium content and maintaining homeostasis [13,43]. However, because ion transport involves many internal mechanisms, we also examined some other possible functions, such as phytohormone treatments and heavy-metal stress. Among these, ABA, auxin, ethylene, and gibberellin play important roles in signaling and response to various stresses such as salinity by influencing physiological mechanisms, activating related proteins, changing root structure, and interacting with each other to regulate plant growth and development [13,43,44,45,46,47,48,49]. Research has also described the functions of *HKT* genes in response to phytohormones in vivo [13,44]. The raise of ethylene levels can increase sodium accumulation in the shoot and salt sensitivity in rice by inducing *OsHKT2;1* [43]. However, the detailed expression patterns of *HKT* genes have been lacking in *B. napus*. In the present study, phytohormones obviously activate the expression of *BnaHKT1;2b*, *BnaHKT1;2a*, *BnaHKT1;3a* and *BnaHKT1;3b* (Figure 7). 

Promoter *cis*-elements play vital roles in environmental adaptation by plants, and analysis of these elements provides a promising method for investigating the possible functions of their associated genes [50]. All of the *BnaHKT* genes have phytohormone, plant development and abiotic response *cis*-elements (Figure 9). Research shows inclusion of ABI4-binding *cis*-element reduces *SlSOS2* expression, decreasing salt resistance in the cultivated tomato [51]. Moreover, *ABRE* and *G-box* elements are favorable binding sites of bZIP TFs that regulate stress responses [52]. *AtbZIP24* and *ABI4* can also negatively regulate *AtHKT1;1*, affecting salt stress tolerance [53]. Therefore, *cis*-elements play crucial roles in stress tolerance. Based on our findings, *BnaHKT* play a role in response to low K^+^ stress and are induced by hormones in response to the stress response, growth, and development.

Heavy-metal pollution has become one of the most important factors restricting crop yield and quality [42]. Considerable evidence supports the idea that *Brassica* species have a high tolerance to heavy metals [54], and *Brassica* species are considered heavy-metal accumulators [35]. In this study, we predicted the potential roles of *BnaHKTs* under heavy-metal stress. Notably, the expression level of *BnaHKTs* could be sensitive to heavy metals in *B. napus*, but they display the functional redundancy in different conditions (Figure 8). Overall, our study systematically studied the characteristics of *HKT* family genes in U-triangle species and documented the expression profiles of *BnaHKT* genes under low K^+^ treatment, phytohormone treatments and heavy-metal stress. These results lay a foundation for further elucidating the function of *BnaHKTs*, and can contribute to improve capacity of the *B. napus* in response to abiotic stresses.

## 4. Materials and Methods

### 4.1. Identification and Annotation of HKT Family Genes

The HKT protein sequences from Arabidopsis, and U-triangle species (*B. rapa*, Bra, genotype AA; *B. nigra*, Bni, genotype BB; *B. oleracea*, Bol, genotype CC; *B. juncea*, Bju, genotype AABB; *B. napus*, Bna, genotype AACC; *B. carinata*, Bca, genotype BBCC) were downloaded from the TAIR database (https://www.arabidopsis.org/ (accessed on 16 November 2022)), the Brassica Database (BRAD, http://brassicadb.cn (accessed on 16 November 2022)) and the *Brassica napus* multi-omics information resource database (BnIR, https://yanglab.hzau.edu.cn/BnIR (accessed on 16 November 2022)) [55]. The *HKT* family genes were predicted using the only known *HKT* gene in *Arabidopsis thaliana*, At4g10310 (*AtHKT1;1*), and the previously studied 29 sequences [41] as queries. NCBI Blastp program (https://blast.ncbi.nlm.nih.gov/Blast.cgi?PROGRAM=blastp&PAGE_TYPE=BlastSearch&LINK_LOC=blasthome (accessed on 24 November 2022)) [56] and Tbtools-BLAST were used to identify the HKT family proteins with default parameters [57]. To examine conserved domains of HKT proteins after BLAST, the National Center for Biotechnology Information (NCBI, https://www.ncbi.nlm.nih.gov/Structure/cdd (accessed on 26 November 2022)) [38] conservative structure domain database (CDD), InterPro [58] online classification of a protein family were used, with an HMM search for proteins containing the TrkH domain (pfam number: PF02386, http://pfam.xfam.org/ (accessed on 26 November 2022)) [59]. The redundant members without the TrkH domain were discarded due to incomplete functions. Eventually, 36 members were identified. Tbtools-Protein Paramter Calc was used to predict the length (number of amino acid residues), molecular weight (MW, in kDa), isoelectric point (pI) and stability index of each HKT protein sequence [57]. Plant Cell—PLoc 2.0—mPLoc (http://www.csbio.sjtu.edu.cn/bioinf/plant-multi/ (accessed on 8 December 2022)) was used to predict the subcellular locations of proteins [60]. 

### 4.2. Phylogenetic and Sequence Analysis of HKT Gene Family Members

MEGA 7.0 (Tokyo Metropolitan University, Tokyo, Japan) was used to build a phylogenetic tree with maximum likelihood method with specific parameters as follows: muscle alignment, Jones–Taylor–Thornton (JTT) model, Bootstrap method 1000, and Partial deletion 80% [61]. Then, evolview (https://www.evolgenius.info/evolview/#/ (accessed on 10 December 2022)) was used to visually refine the phylogenetic tree for presentation [62]. 

To predict the presence of transmembrane helices in the HKT family proteins in the six *Brassica* species, the online software DeepTMHMM Server (https://dtu.biolib.com/DeepTMHMM (accessed on 11 January 2022)) was used [11,63]. To analyze sequences in Arabidopsis and the U-triangle species, the ClustalW software (Kyoto University Bioinformatics Center) (https://www.genome.jp/tools-bin/clustalw (accessed on 3 February 2022)) was used, with general settings parameters as follows: fast/approximate pairwise alignment, clustal output format [64]. The ClustalW results were visualized by ESPript 3.0 (https://espript.ibcp.fr/ESPript/cgi-bin/ESPript.cgi (accessed on 3 February 2022)) [65]. Then, the sequence logos of the four P-loops were generated from WebLogo Version 2.8.2 (http://weblogo.berkeley.edu/logo.cgi (accessed on 4 February 2022)) [66]. The 2000-bp regions 5’ to the translation start sites of each *HKT* gene were acquired from the *Brassica napus* L. information resource (BnIR) as a promoter sequence by Tbtools [55,57], and the *cis*-elements were analyzed using the PlantCARE website (http://bioinformatics.psb.ugent.be/webtools/plantcare/html/ (accessed on 3 February 2022)) [11,67].

### 4.3. Structural Analysis of Conserved Motifs and Genes of the HKT Family Members

The online program Multiple Expectation Maximization for Motif Elucidation (MEME) (https://meme-suite.org/meme/doc/meme.html (accessed on 1 February 2022)) was used to identify and analyze the conserved motif of the HKT family members [68]. The specific parameters were as follows: zero or one occurrence per sequence, maximum number of motifs 5; and optimum width of each motif between 6 wide and 50 wide (inclusive) [69]. The motif structure and gene structure are shown together with the evolutionary tree using the Gene Structure View in TBtools software (https://github.com/CJ-Chen/TBtools (accessed on 1 February 2022)) [57].

### 4.4. Chromosomal Localization and Colinearity Analysis of HKT Genes

The chromosomal location information of *HKT* family genes was extracted using the genome sequence annotation of *Brassica* species (BnIR, https://yanglab.hzau.edu.cn/BnIR (accessed on 16 November 2022)) [55]; the *HKT* family genes were mapped to their corresponding chromosomes by using MapChart V2.32 [70]. Moreover, tandem repeats were identified based on the physical location of *HKT* tandem repeats on a single chromosome. To better understand the colinearity relationship, TBtools-One Step MCScanX and TBtools-Amazing Super Circos were used to analyze the homologous genetic relationships among *HKT* genes [57]. Then, the nonsynonymous substitution rates (Ka), synonymous substitution rates (Ks) and Ka/Ks ratios were calculated to estimate selection pressures during evolution. 

### 4.5. Expression Profile Analysis of HKT Genes

The rapeseed cultivar Zhongshuang 11 (ZS11) was used as the plant material for potassium and hormone treatments (IAA, GA3, 6-BA, ABA, and ACC). The plant materials and treatments were as described previously [71], and RNA-Seq data were obtained from BnaGADB v1.0 (http://www.bnagadb.cn/ (accessed on 2 January 2022)) to investigate the phytohormone-responsive expression patterns of *BnaHKTs*. For heavy-metal treatments, 15 mg/L As and 30 mg/L Cd were used as a most optimal concentration due to the previous study [33]. The healthy seeds were selected from P163 and P087 and treated with distilled water (the control) and 15 mg/L As or 30 mg/L Cd. The relative expression levels of all *BnaHKT* genes were calculated using TopHat and Cufflinks, and normalized into FPKM values (fragments per kilobase of transcript per million mapped reads) [72,73]. Finally, to better display the data, the data were converted into Log_10_ (FPKM value + 1) followed by TBtools [57] to generate the heatmaps to show *BnaHKT* expression. The RNA-seq datasets, calculation methods and so on were as described elsewhere [72].

### 4.6. RT-qPCR Analysis of BnaHKT Genes

Total RNA extraction and RT-qPCR analysis are the same as described in our previous research [33]. The qualified RNA was reverse transcribed into complementary DNA (cDNA) with an RNA PCR Kit (AMV) Ver. 3.0 (Takara, Dalian, China). The subsequent qPCR analysis was performed using SYBR Premix Ex Taq II (Takara, Dalian, China) on a Bio-Rad CFX96 Real Time System (Bio-Rad Laboratories, Hercules, CA, USA) as previously described [33]. Finally, the results were normalized to the reference gene *BnaActin7* (EV116054) [74] via the 2^−∆∆Ct^ method [75]. Error bars represent standard errors from three independent biological replicates in this study. The primers are listed in Table S1. 

## 5. Conclusions

In this study, we identified 36 *HKT* family genes in the six *Brassica* U-triangle species, and classified them into four subgroups according to their evolutionary relationships. Most of the *HKT* family genes experienced strong purifying selective pressure after duplication. On the basis of *BnaHKTs* transcript levels, functional diversity is displayed: *BnaHKT1;1a* and *BnaHKT1;1b* induced by low K^+^, *BnaHKT1;2a*, *BnaHKT1;2b*, *BnaHKT1;3a* and *BnaHKT1;3b* sensitive to phytohormones. Furthermore, most *BnaHKTs* were strongly suppressed by heavy-metal stress. Our findings provide novel clues for future studies on the potential roles of *BnaHKTs* in response to heavy-metal stress. In summary, these results provided valuable information to further explore the functional *BnaHKTs*, and improve the tolerance to heavy-metal stress and other abiotic stresses in *Brassica* species.

## Figures and Tables

**Table 1:** Characteristics of HKTs from Arabidopsis and the *Brassica* U-triangle species.

Protein Name	Gene ID	Length (aa)	MW (kDa)	pI	Predicted Subcellular Location
AtHKT1;1	AT4G10310	506	57.45	8.86	Cell membrane
BraHKT1;1	BraA02g030010.3.5C.1	507	58.02	8.84	Cell membrane
BraHKT1;2	BraA03g027520.3.5C.1	564	63.27	9.61	Cell membrane
BraHKT1;3a	BraA09g029230.3.5C.1	350	39.60	9.19	Cell membrane
BraHKT1;3b	BraA09g029230.3.5C.2	496	56.12	9.42	Cell membrane
BraHKT1;4	BraA09g029260.3.5C.1	359	41.09	9.44	Cell membrane
BniHKT1;1	BniB05g058650.2N	506	57.60	8.97	Cell membrane
BniHKT1;2	BniB08g035830.2N	529	59.79	9.63	Cell membrane
BniHKT1;3	BniB08g052410.2N	496	56.22	9.34	Cell membrane
BniHKT1;4	BniB08g052460.2N	356	40.62	9.55	Cell membrane
BolHKT1;1	Bol020851	507	57.94	9.01	Cell membrane
BolHKT1;2	Bol025669	506	57.40	9.66	Cell membrane
BolHKT1;3	Bol007718	447	50.62	9.06	Cell membrane
BjuHKT1;1a	BjuVB05G47650	506	57.60	8.97	Cell membrane
BjuHKT1;1b	BjuVA02G31630	507	58.05	8.93	Cell membrane
BjuHKT1;2a	BjuVB08G29360	506	57.52	9.72	Cell membrane
BjuHKT1;2b	BjuVA03G28370	585	65.83	9.76	Cell membrane
BjuHKT1;3a	BjuVB08G41970	350	39.70	9.02	Cell membrane
BjuHKT1;3b	BjuVA09G28320	496	56.26	9.48	Cell membrane
BjuHKT1;4a	BjuVB08G41990	500	57.11	9.62	Cell membrane
BjuHKT1;4b	BjuVA09G28330	503	57.57	9.45	Cell membrane
BnaHKT1;1a	BnaA02T0271600ZS	507	58.02	8.84	Cell membrane
BnaHKT1;1b	BnaC02T0368700ZS	507	57.97	9.01	Cell membrane
BnaHKT1;2a	BnaC03T0301900ZS	503	57.14	9.62	Cell membrane
BnaHKT1;2b	BnaA03T0254300ZS	585	65.84	9.76	Cell membrane
BnaHKT1;3a	BnaA09T0257800ZS	496	56.28	9.52	Cell membrane
BnaHKT1;3b	BnaC09T0303500ZS	509	57.58	9.5	Cell membrane
BnaHKT1;4a	BnaC09T0303900ZS	522	59.89	9.63	Cell membrane
BnaHKT1;4b	BnaA09T0258000ZS	430	49.11	9.1	Cell membrane
BcaHKT1;1a	BcaB05g23910	506	57.63	9.04	Cell membrane
BcaHKT1;1b	BcaC03g16406	507	57.97	9.01	Cell membrane
BcaHKT1;2a	BcaB01g02755	523	58.74	9.37	Chloroplast
BcaHKT1;2b	BcaC01g04681	506	57.40	9.66	Cell membrane
BcaHKT1;3a	BcaB01g01466	496	56.22	9.34	Cell membrane
BcaHKT1;3b	BcaC04g21028	496	56.16	9.52	Cell membrane
BcaHKT1;4a	BcaB01g01465	500	57.07	9.6	Cell membrane
BcaHKT1;4b	BcaC04g21025	503	57.53	9.45	Cell membrane

At, *A. thaliana*; Bra, *B. rapa*; Bni, *B. nigra*; Bol, *B. oleracea*; Bju, *B. juncea*; Bna, *B. napus*; Bca, *B. carinata*, aa, amino acid residues; pI, isoelectric point.

## Data Availability

All other datasets supporting the results of this article are included within the article and Supplementary Materials.

## Supplementary Materials

The following supporting information can be downloaded at https://www.mdpi.com/article/10.3390/plants12213768/s1. Table S1: Primers used for RT-qPCR analysis of eight *BnaHKT* genes.
